# The balance between the serum levels of IL-6 and IL-10 cytokines discriminates mild and severe acute pneumonia

**DOI:** 10.1186/s12890-016-0324-z

**Published:** 2016-12-01

**Authors:** Rita de Cássia Coelho Moraes de Brito, Norma Lucena-Silva, Leuridan Cavalcante Torres, Carlos Feitosa Luna, Jaílson de Barros Correia, Giselia Alves Pontes da Silva

**Affiliations:** 1 Institute of Integral Medicine Professor Fernando, Figueira (IMIP), Pediatrics, Rua Dona Benvinda de Farias 159, apt 1101, Boa Viagem, Recife, Pernambuco Brazil; 2 Institute of Integral Medicine Professor Fernando Figueira (IMIP), Oncology and Aggeu Magalhães Research Center, Fiocruz-PE, Immunology, Recife, Brazil; 3 Institute of Integral Medicine Professor Fernando, Figueira, IMIP, Translational Medicine Laboratory, Recife, Brazil; 4 Aggeu Magalhães Research Center, Fiocruz-PE, Public Health, Recife, Brazil; 5 Federal University of Pernambuco, Pediatrics, Recife, Brazil

**Keywords:** Pneumonia, Inflammation, Cytokines, Immune response

## Abstract

**Background:**

To identify markers for earlier diagnosis of severe pneumonia, we assess the correlation between serum cytokine profile of children with different pneumonia severity.

**Methods:**

In 25 hospitalized children, 7 with mild pneumonia and 18 with severe pneumonia, the serum concentration of 11 cytokines in three sampling times were dosed. Statistical analysis included parametric and non-parametric tests, Pearson correlation and ROC curve for cut-off definition of cytokines.

**Results:**

At admission, IL-6 serum levels were high in mild or severe pneumonia, and was associated to vomiting (*P* = 0.019) in both groups; and also to dyspnea (*P* = 0.012) and white blood cell count (*P* = 0.045) in patients with severe pneumonia. IL-10 levels were also high in patients with pneumonia and were associated to lymphocytosis (P = 0.025). The ROC curve of the IL-6:IL-10 serum levels ratio discriminated severe pneumonia cases at admission, and persistence of infection in the third day of antibiotic therapy, with positive predictive values of 93% and 89%, respectively.

**Conclusions:**

The balance between IL-6 and IL-10 serum levels showed to be a more discriminative marker for severity definition and evaluation of recovery in patients with pneumonia.

**Electronic supplementary material:**

The online version of this article (doi:10.1186/s12890-016-0324-z) contains supplementary material, which is available to authorized users.

## Background

Community acquired pneumonia (CAP) is a serious illness of the lower respiratory tract, responsible for high morbidity in children, especially those younger than five years old [1–3]. The World Health Organization (WHO) uses clinical manifestations as a parameter for definition of severity ratings (pneumonia and severe pneumonia) [4]. The gradient of clinical severity arises from a complex interaction between environmental factors, pathogenicity of the etiologic agent and host characteristics, including immunological competence, malnutrition, anemia and other comorbidities [5–13]. Clinical data associated to blood count and serum level of the C-reactive protein (CRP) are determinants for therapeutic management of acute respiratory disease in clinical practice [3, 14–16]. Blood count and serum levels of CRP are low-cost laboratory tests, accessible in health services in countries with limited resources and their results help the differentiation between viral or bacterial respiratory infection [3, 14, 17]. High levels of CRP are caused bythe activity of circulating tumor necrosis factor (TNF) in the liver as a response to the infectious process; therefore, CRP is considered a nonspecific marker [18].

Recently, serum and bronchoalveolar levels of cytokines have been related to severity of pulmonary inflammatory process [9, 19–21]. During the early stages of pneumonia, the alveolar macrophages produce a variety of cytokines and inflammatory chemokines, which attract and activate polymorphonuclear leukocytes that will mount an appropriate immune response in the lung parenchyma [22–24]. Circulating levels of TNF, Interleukin (IL)-1, IL-6, IL-8, IL-12 and interferon-gamma (IFN-γ) are found in patients with CAP [7, 9, 19–21]. The cytokines IL-1, TNF and IL-6 are important for the acute phase response [7, 9, 25], while modulation of inflammatory responses of the airways is related to the expression of the anti-inflammatory cytokines,IL-10 and IL-4 [6, 8, 24]. Studies on the inflammatory process during acute respiratory disease have reported conflicting results. Most studies that seek to relate the severity of acute respiratory disease with cytokines serum levels have evaluated cytokine levels in a single time point and samples were collected from adult patients, who may have different inflammatory responses from those observed in children due to the anatomical-functional differences of the pulmonary alveoli, in addition to the immune system immaturity [7, 9–13, 21, 26]. The aim of the present study was to evaluate the correlation between the cytokines profile in the serum of children and adolescents with different pneumonia severity, defined by clinical manifestations and alterations in the blood count, in order to identify markers for early diagnosis of severe pneumonia.

## Methods

### Design and the subjects of the study

This was an observational, prospective and exploratory study, which investigated the association between serum cytokines levels with pneumonia severity. The classification of clinical severity was based on the criteria defined by WHO and established in the Brazilian Guidelines for CAP in Pediatrics [3]. Briefly, cough or tachypnea (respiratory rate ≥ 50 breaths per minute in an infant or ≥ 40 breaths per minute in one year or older children) with radiological evidence of pulmonary consolidation was defined as pneumonia. We considered severe pneumonia either the presence of chest indrawing in children with cough or difficult breathing, which were criteria for severe pneumonia by WHO, or presence of severe respiratory distress or inability to drink or central cyanosis in a child with cough or difficult breathing, which are criteria for classification of a very severe pneumonia by WHO. Cytokine levels of children with pneumonia were compared with cytokine levels of children with severe pneumonia.

The patients evaluated in the present study were admitted to three hospitals (Hospital da Restauração, Hospital Helena Moura and the Instituto de Medicina Integral Professor Fernando Figueira—IMIP) in the municipality of Recife, northeastern Brazil, from June to December 2012. All three hospitals belong to the network of public health assistance of the Brazilian Health System (Sistema Único de Saúde—SUS).

The exclusion criteria were age under six months, previous history of immunodeficiency, clinical suspicion of tuberculosis, heart disease, neurological disease, cystic fibrosis, bronchiolitis obliterans and severe persistent asthma. Children that were transferred from other hospitals, and were using antibiotics therapy for more than 3 days at the admission were also excluded, because we were not able to collect biological samples for analysis in two of three time points defined in the research protocol.

The present study was approved by the Research Ethics Committees of the IMIP hospital (protocol 2886) and the Hospital da Restauração (protocol CAAE-0014.0.102.000-11). A clearance letter was also issued by the Health Secretary of the state of Pernambuco authorizing the research implementation in the Hospital Helena Moura. Data collection was performed after obtaining written Informed Consent signed by the children’s legal guardian.

### Definition of variables

A form containing demographic (age, gender, place of residence, socio-economic conditions) and clinical questions (duration of the disease, general symptoms and related acute respiratory disease, including fever, coughing, difficult breathing, subcostal recession, cyanosis and nasal flaring, respiratory frequency, heart rate and oxygen saturation) was filled out at hospital admission for each patient. Presence of complications, such as pleural effusion, respiratory insufficiency and anemia were also investigated. Hemoglobin levels lower than 11,11.5 or 12 g/dL defined anemia for children less than five years old, children 5 to 11 years old and adolescents 12 to 14 years old, respectively [27]. Oxygen saturation and heart rate were evaluated in patients with no oxygen supply using the Rossmax Pulse Oximeter SB100. Respiratory frequency was defined as the number of breaths per minute.

Chest x-ray and blood tests were performed at hospital admission. Blood samples were collected for cytokine dosage in three sampling times: on the day of admission (D0) before any medical intervention; three days after the therapeutic scheme had started (D3), when a clinical improvement of 80% in children treated with proper antibiotics is expected; and eight days (D8) after the therapeutic scheme had started, when complete illness resolution is expected.

### Cytokines analysis

Peripheral blood samples were collected and sent to the laboratory within 40 min. The cytokines IL-8, IL-1β, IL-6, IL-10, TNF, IL-12p70 and IL-17A, IL-2, IL-4, IFN-γ were evaluated by the BD™ Cytometric Bead Array Human Inflammation reagents and the Human Th1/Th2/Th17 Cytokine kit. These tests were chosen because they allow the quantification of several cytokines simultaneously using a small sample volume. The analysis of IL-5 expression was performed by immunoassay using the BD OptEIA® Human IL-5 reagent, as recommended by the manufacturer (Becton Dickinson, Franklin Lakes, New Jersey). Of 25 children, cytokines serum levels were determined in 23 at admission, and 17 on D3 and D8 of treatment.

### Data analysis and statistics

Numerical variables, including age, respiratory and heart frequency and oxygen saturation among others, were subjected to the normality test of Kolmogorov-Smirnov, and those that did not present normal distribution were expressed by median, minimum and maximum values. The median difference was assessed by Mann–Whitney U non-parametric test. Categorical data were analyzed using absolute and relative frequency tables and comparative analysis of the two studied groups (pneumonia and severe pneumonia) was performed by the Chi-square test or the Fisher’s 2-tailed test,when indicated. The non-parametric Friedman test with its respective post-hoc was used for intra-group analysis. A significance level of 5% was considered in all analyzes.

The correlation between cytokine levels, clinical variables and blood count were evaluated by Pearson correlation coefficient, except for the subcostal recession variable, which was analyzed by the Spearman’s coefficient. The ROC curve, area under the curve, sensitivity, specificity, positive and negative predictive value were used to find the cut-offs for the IL6 and IL6:IL10 ratio levels. The statistical analyzes and graphics were performed using the *Action* 26 Excel software (Microsoft, Albuquerque, New Mexico) and GraphPad Software Inc. (La Jolla, California).

## Results

### Patient characterization

Of the 25 children evaluated in the present study, 7 were diagnosed with pneumonia and 18 with severe pneumonia. Almost all patients had an updated and complete vaccination scheme (92%), were breast fed (88%) and had adequate weight for age (96%). Three patients (12%) lived with more than seven people in the same household, most patients (80%) were from the countryside of the state of Pernambuco, and a third (32%) of the respondents reported the presence of smokers at home. Low maternal schooling was common among the children’s legal guardians (96%).

Severe pneumonia was more frequent among children younger than 5 years old (*P* = 0.027), and there was no difference in disease severity between gender (*P* = 0.656). Coughing and fever were observed in all patients of both groups. However, the duration of fever (*P* = 0.000) and anorexia (*P* = 0.023) during hospitalization were longer in severe patients. The most frequent symptoms in patients with severe pneumonia were subcostal recession (*P* < 0.000), difficult breathing (*P* = 0.007), O_2_ saturation (*P* = 0.011), wheezing (*P* = 0.032) and vomiting (*P* = 0.021) (Additional file 1: Table S1).

Anemia occurred in 61.1% of the patients with severe pneumonia and leukocytosis was observed in both studied groups. The median leukocyte count was higher in the pneumonia group than in children with severe pneumonia, but with no statistical difference between the two groups. Patients with severe pneumonia were diagnosed later (median of 10 days) than children with pneumonia (median of 7 days) (*P* = 0.023).

Of the 18 patients with severe pneumonia, 11 (61%) needed chest drainage for a median of 11 days (range 4 to 29 days). Biochemical analysis and pleural fluid culture were not performed in any of the cases. In nine patients with severe pneumonia (50%), oxygen was supplied by a Venturi mask for a median of 7 days.

### Cytokine profile of patients with pneumonia and severe pneumonia

Serum circulating levels of TNF, IL-1β, IL-8, IL-17 and IL-5 in the three sampling times were similar for both studied groups. At diagnosis, IL-10 levels were lower (3.68 *vesus* 1.99, *P* = 0.074), and IL-6 levels were higher (44.07 *versus* 19.98, *P* = 0.155) in children with severe pneumonia than in children with non-severe pneumonia; however, no statistical difference was observed between the two groups. Furthermore, IL-6 serum levels significantly reduced in patients with pneumonia (*P* = 0.041) and severe pneumonia (*P* = 0.001) during recovery (Table 1).

**Table 1:** Cytokine profiles of patients with pneumonia according to severity and disease duration

	Severe pneumonia				Non-severe pneumonia				Severe x non-severe pneumonia
Cytokines (days)	Q_1_	Q_3_	Median	*P-value	Q_1_	Q_3_	Median	**P-value	***P-value
IL10 (D0)	1.50	2.68	1.99		2.44	4.05	3.68		0.074
IL10 (D3)	1.35	3.59	1.92		1.41	6.12	2.31		0.441
IL10 (D8)	1.99	5.55	2.59	0.417	1.72	11.43	2.16	0.549	1.000
IL6 (D0)	26.62	108.47	44.07		13.55	445.55	19.98		0.155
IL6 (D3)	9.27	40.48	16.45		4.95	56.34	11.13		0.959
IL6 (D8)	3.27	15.32	8.41	**0.001**	2.98	12.66	4.94	**0.041**	0.442

Note

*P-value is related to the difference in serum cytokines levels in D0, D3 and D8 for children with severe pneumonia

**P-value is related to the difference in serum cytokines levels in DO, D3 and D8 for children with non-severe pneumonia (intragroup analysis considered children with all three samples evaluated)

***P-value is related to comparisons of serum cytokine levels between group of children with severe and non-severe pneumonia in each time point D0, D3 or D8 (Intergroup analtsis considered all samples collected for each time point). Q1, first quartil, and Q3, thrird quartil. *P*-value ≤ 0.05 was considered significant, significant values are shown in bold

There was a correlation between some cytokines and the clinical signs of severity and nonspecific symptoms related to the infection. In patients with severe pneumonia and pneumonia, IL-6 levels were associated with vomiting; however, in the severe pneumonia group, IL-6 levels were also associated to dyspnea, suggesting that this cytokine plays a role in pneumonia severity (Table 2).

**Table 2:** Correlation between clinical signs and blood count with cytokines serum levels of patients with severe and non-severe pneumonia

	IL-12p70		TNF		IL-10		IL-6		IL1β		IL-8		IFN		IL-17A		IL5		IL-6/IL-10	
	r	*P*	r	*P*	r	*P*	r	*P*	r	*P*	r	*P*	r	*P*	r	*P*	r	*P*	r	*P*
Severe pneumonia																				
Clinical signs (days)																				
Abdominal pain	0.30	0.263	0.06	0.812	0.03	0.923	0.05	0.844	0.38	0.147	0.32	0.229	0.07	0.803	0.78	0.799	−0.37	0.150	0.04	0.897
Sickness	−0.03	0.906	−0.11	0.676	−0.09	0.742	0.03	0.902	−0.08	0.759	−0.12	0.661	−0.47	0.093	−0.42	0.153	−0.26	0.306	0.05	0.842
Fever	0.33	0.208	0.25	0.359	0.28	0.293	0.46	0.073	0.35	0.190	−0.13	0.644	−0.29	0.315	−0.38	0.196	−0.36	0.155	0.43	0.097
Anorexia	−0.07	0.795	−0.04	0.874	−0.24	0.374	0.30	0.263	−0.43	0.097	−0.09	0.731	−0.26	0.377	−0.19	0.530	0.08	0.766	0.31	0.238
Dyspnea	−0.03	0.905	−0.02	0.945	−0.12	0.654	**0.61**	**0.012**	0.34	0.196	0.27	0.308	−0.09	0.761	−0.2	0.523	−0.39	0.126	**0.62**	**0.010**
Subcostal recession	0.15	0.568	0.14	0.600	−0.14	0.605	0.03	0.918	0.36	0.166	−0.08	0.757	**0.53**	**0.053**	−0.15	0.615	−0.05	0.842	0.20	0.467
Difficult breathing	−0.10	0.715	0.09	0.736	−0.21	0.443	−0.05	0.862	0.09	0.738	0.23	0.388	−0.12	0.692	−0.17	0.581	0.18	0.484	−0.01	0.980
Chest pain	−0.18	0.518	0.01	0.971	−0.21	0.443	0.08	0.785	−0.12	0.676	−0.28	0.311	−0.23	0.454	−0.33	0.264	−0.03	0.912	−0.17	0.540
Coughing	−0.12	0.667	−0.02	0.532	−0.43	0.095	0.06	0.827	−0.44	0.090	−0.34	0.199	−0.39	0.165	−0.43	0.145	−0.35	0.167	0.11	0.688
Vomiting	−0.31	0.211	−0.24	0.376	0.16	0.549	**0.58**	**0.019**	−0.12	0.669	−0.08	0.761	0.22	0.447	0.28	0.362	−0.02	0.939	**0.55**	**0.028**
Blood count																				
Hemoglobin	−0.06	0.814	−0.18	0.517	−0.16	0.556	0.18	0.516	−0.12	0.662	**−0.62**	**0.011**	−0.03	0.919	−0.25	0.417	−0.35	0.167	0.18	0.513
Leukocytes	−0.34	0.204	−0.25	0.357	−0.11	0.674	**0.51**	**0.045**	−0.08	0.779	−0.24	0.371	0.4	0.152	0.31	0.309	−0.28	0.277	**0.52**	**0.037**
Hemacias	−0.29	0.275	−0.41	0.114	−0.15	0.57	0.08	0.757	−0.27	0.306	−0.23	0.402	−0.07	0.809	−0.05	0.866	−0.44	0.081	0.08	0.762
Neutrophils	−0.09	0.753	−0.15	0.583	−0.25	0.351	0.43	0.1	−0.32	0.228	−0.37	0.164	−0.27	0.357	−0.29	0.346	−0.21	0.419	0.44	0.088
Eosinophils	−0.07	0.805	−0.09	0.755	−0.1	0.735	−0.13	0.639	0.23	0.413	−0.48	0.069	−0.18	0.552	−0.16	0.616	0.35	0.18	−0.10	0.720
Lymphocytes	0.26	0.323	0.37	0.157	0.32	0.229	−0.33	0.218	0.45	0.084	0.46	0.071	0.32	0.268	0.31	0.307	0.4	0.114	−0.33	0.212
Monocytes	0.34	0.204	**0.56**	**0.025**	0.32	0.223	−0.29	0.282	**0.52**	**0.04**	0.26	0.332	0.16	0.587	0.07	0.819	0.02	0.954	−0.29	0.279
Platelets	0.01	0.963	0.11	0.673	−0.09	0.746	−0.21	0.431	−0.07	0.807	0.37	0.157	−0.02	0.955	0.06	0.84	0.42	0.09	−0.20	0.466
Non-severe Pneumonia																				
Clinical signs (days)																				
Abdominal pain	**0.91**	**0.012**	**0.89**	**0.019**	**0.81**	**0.049**	0.35	0.503	**0.81**	**0.054**	0.40	0.430	−0.38	0.534	−0.56	0.443	−0.38	0.406	0.34	0.514
Sickness	0.29	0.579	0.29	0.287	0.41	0.418	−0.62	0.19	−0.30	0.569	−0.54	0.273	−0.31	0.612	−0.92	0.077	0.38	0.401	−0.62	0.185
Fever	0.53	0.283	0.17	0.748	0.71	0.112	0.06	0.912	0.22	0.680	−0.06	0.909	0.11	0.856	0.10	0.897	−0.21	0.659	−0.17	0.745
Anorexia	−0.08	0.887	0.32	0.538	−0.02	0.975	0.36	0.479	0.57	0.237	0.30	0.561	−0.17	0.781	−0.28	0.722	−0.25	0.591	0.11	0.832
Dyspnea	−0.12	0.822	−0.23	0.658	0.19	0.722	−0.10	0.852	−0.18	0.739	−0.23	0.666	−0.24	0.693	−0.32	0.681	0.29	0.535	−0.11	0.837
Subcostal recession	-	-	-	-	-	-	-	-	-	-	-	-	-	-	-	-	-	-	-	-
Difficult breathing	−0.13	0.809	0.32	0.543	−0.12	0.824	−0.19	0.371	0.18	0.739	−0.17	0.744	−0.38	0.534	−0.38	0.616	0.33	0.465	−0.20	0.707
Chest pain	-	-	-	-	-	-	-	-	-	-	-	-	−0.25	0.685	-	-	0.28	0.550	-	-
Coughing	0.31	0.555	0.38	0.454	0.43	0.400	−0.48	0.340	−0.15	0.781	−0.39	0.450	−0.48	0.417	**−0.95**	**0.046**	0.46	0.300	−0.48	0.333
Vomiting	−0.09	0.884	0.13	0.839	−0.21	0.731	**1.00**	**<0.001**	0.73	0.164	**0.99**	**0.001**	-	-	-	-	−0.58	0.229	**1.00**	**<0.001**
Blood count																				
Hemoglobin	0.62	0.189	0.24	0.641	0.54	0.27	0.3	0.57	0.42	0.402	0.23	0.66	**0.97**	**0.006**	**0.97**	**0.031**	**−0.82**	**0.024**	0.29	0.575
Leukocytes	−0.55	0.256	−0.35	0.496	−0.68	0.138	0.49	0.328	0.16	0.765	0.44	0.387	0.54	0.348	0.72	0.282	−0.48	0.273	0.49	0.319
Hemacias	−0.45	0.371	−0.56	0.251	−0.65	0.166	0.29	0.571	−0.23	0.666	0.31	0.552	0.71	0.178	0.74	0.256	−0.49	0.268	0.31	0.551
Neutrophils	−0.64	0.172	−0.21	0.688	−0.76	0.078	0.49	0.321	0.2	0.699	0.5	0.311	−0.01	0.987	0.1	0.9	−0.18	0.707	0.50	0.313
Eosinophils	0.49	0.325	0.19	0.714	0.71	0.113	−0.38	0.455	−0.13	0.813	−0.43	0.399	−0.31	0.61	−0.4	0.597	0.33	0.467	−0.39	0.440
Lymphocytes	0.73	0.097	0.47	0.346	**0.87**	**0.025**	−0.48	0.333	−0.01	0.986	−0.47	0.345	−0.45	0.443	−0.62	0.379	0.3	0.516	−0.49	0.320
Monocytes	0.08	0.888	−0.24	0.646	0.36	0.483	−0.44	0.386	−0.39	0.448	−0.56	0.247	0.02	0.98	−0.09	0.913	0.23	0.623	−0.45	0.374
Platelets	−0.42	0.411	−0.4	0.438	−0.34	0.506	−0.48	0.338	−0.71	0.115	−0.4	0.43	−0.39	0.519	−0.56	0.439	0.7	0.08	−0.47	0.346

Note: r, Person correlation. *P-value* <0.05 was considered significant, significant values are shown in bold

In patients with pneumonia at hospital admission, IL-10 was associated to lymphocytosis (*P* = 0.025), and in patients with severe pneumonia, the proliferation of defense cells was demonstrated by the positive association between IL-6 levels and leukocyte count (*P* = 0.045) and the levels of TNF (*P* = 0.025) and IL-1β (*P* = 0.040) with monocytosis (Table 2).

### Pneumonia recovery and cytokines profile

Oxygen saturation, temperature, respiratory and cardiac frequencies are predictive paramenters for pneumonia severity, and were evaluated at admission, on D3 and D8 of hospitalization for all children (Additional file 2: Table S2).

At admission, O_2_ saturation was the most important criterion of severity (94% for severe pneumonia *versus* 98% for non-severe pneumonia, *P* = 0.011), but no significant differences were found in temperature and respiratory or cardiac frequencies. On day D3 of hospitalization, besides the O_2_ saturation difference (*P* = 0.021), it was possible to distinguish severe pneumonia from non-severe cases based on respiratory frequency (*P* = 0.030) and persistence of fever (*P* = 0.000). On D8 of hospitalization, O_2_ saturation and persistence of fever were not predictive parameters for pneumonia severity, but high respiratory frequency still predicted disease severity (*P* = 0.008).

Considering data of children who had all three samples (D0, D3 and D8) collected, we performed a longitudial analysis for temporal evaluation of the clinical signs recovery. Children with non-severe pneumonia had significant temperature improvement on D3 (*P* = 0.002). On the other hand, in patients with severe pneumonia, no statistical difference was observed from D0 to D3 (*P* = 0.393), but a significant temperature change occurred from D3 to D8 (*P* = 0.001). The improvement of the respiratory frequency was also faster in patients with non-severe pneumonia than in patients with severe pneumonia. In non-severe pneumonia group, a significant difference of the respiratory frequency occurred from D0 to D3 (*P* = 0.012), while in patients with severe pneumonia, the difference was only noticeable on D8 in comparison with D3 (*P* = 0.014) or D0 (*P* = 0.001) (Additional file 2: Table 2).

**Fig. 1:**
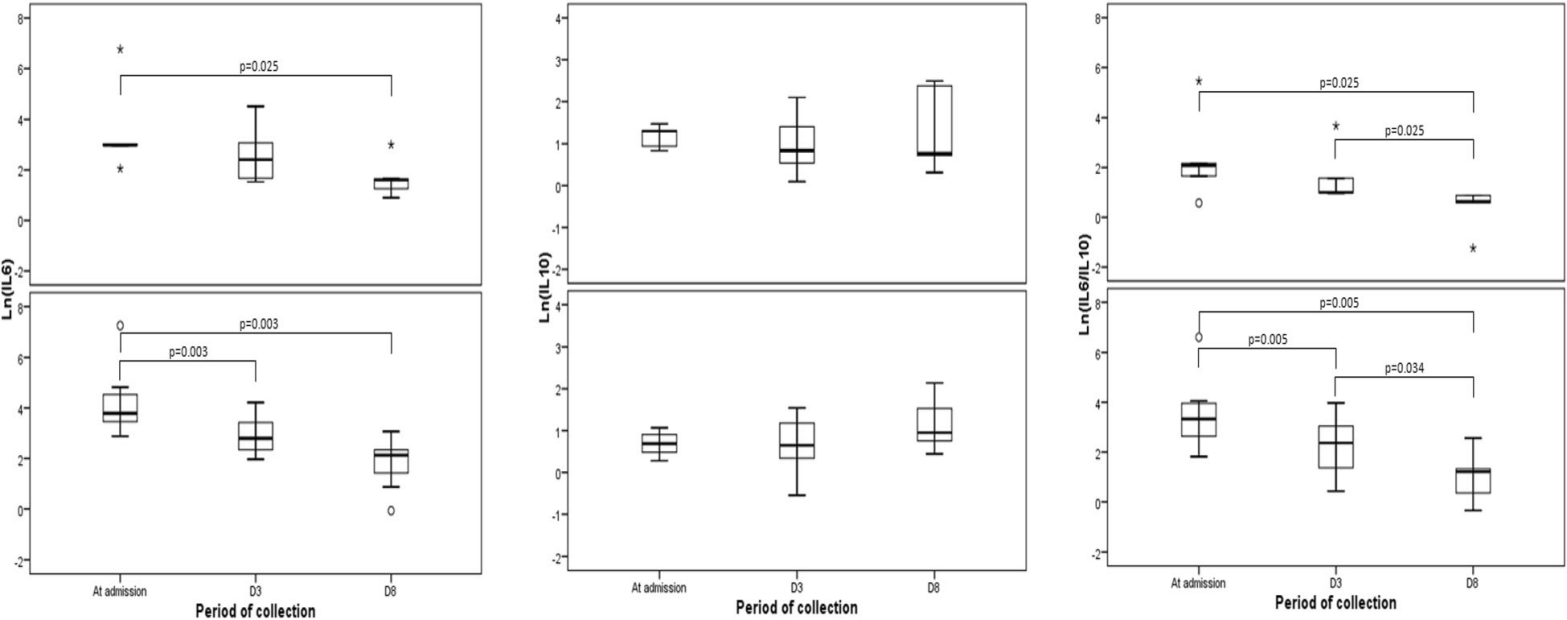
Balance between IL-6 and IL-10 during recovery from pneumonia of different severity. Seric levels of IL-6 (left) and IL-10 (middle) cytokines, and the ratio of seric levels of IL-6:IL-10 (right) in group of children diagnosed with non-severe pneumonia (top) or severe pneumonia (bottom). IL-6 levels, in non-severe pneumonia, at admission x D8 (*P* = 0.025); IL-6 levels, in severe pneumonia, at admission x D3 (*P* = 0.003) and at admission x D8 (*P* = 0.003); Ratio IL-6:IL-10, in non-severe pneumonia, at admission and D3 (*P* = 0.025) and D3 x D8 (*P* = 0.025); Ratio IL-6:IL-10, in severe pneumonia, at admission x D3 (*P* = 0.005); at admission and D8 (*P* = 0.005) and D3 x D8 (*P* = 0.034)

Regarding the cytokine levels, the reduction of IL-6 serum levels during recovery of children with severe pneumonia was the effect more pronuanced (Fig. 1). The ratio between the median of IL-6 and IL-10 serum levels in patients with severe and non-severe pneumonia was respectively 22 and 5 at admission, but 9 and 5 on D3, and 3 and 2 on D8 of antibiotics therapy.

According to the ROC curve analysis, at hospital admission, IL-6 serum levels equal or higher than 21.1 pg/mL and IL-6:IL-10 ratio of at least 9.61 may discriminate severe pneumonia from mild disease with a sensitivity of 76.5%, corresponding to a positive predictive value of 93% (Table 3). Furthermore, in the third day of antibiotics therapy, IL6:IL10 serum levels higher than 5.0 predict persistence of the symptoms and may help medical decision in adjusting the therapeutics for severe cases, and discharge mild pneumonia cases. The decrease in the IL-6:IL-10 ratio to 3 in patients with severe pneumonia on D8 coincided with the temperature and respiratory frequency recovery to normal levels.

**Table 3:** Interleukin (IL)-6 and the IL-6:IL-10 serum levels ratio as marker of pneumonia severity and recovery

IL-6					IL-6/IL-10				
Time-points	Severe pneumonia	Non-severe pneumonia		CI 95%	Time-points	Severe pneumonia	Non-severe pneumonia		CI 95%
At admission			Sen = 76.5	49.8 – 92.2	At admission			Sen = 76.5	49.8 – 92.2
>21.1	13	1	Spec = 83.3	36.5 – 99.1	>9.61	13	1	Spec = 83.3	36.5 – 99.1
≤21.1	4	5	PPV = 92.9	64.2 – 99.6	≤9.61	4	5	PPV = 92.9	64.2 – 99.6
			NPV = 55.6	22.7 – 84.7				NPV = 55.6	22.7 – 84.7
D3			Sen = 58.3	28.6 – 83.5	D3			Sen = 72.7	39.3 – 92.7
>11.28	7	2	Spec = 60.0	17.0 – 92.7	>5.00	8	1	Spec = 80.0	29.9 – 98.9
≤11.28	5	3	PPV = 77.8	40.2 – 96.1	≤5.00	3	4	PPV = 88.9	50.7 – 99.4
			NPV = 37.5	10.2 – 74.1				NPV = 57.1	20.2 – 88.2
D8					D8				
>5.07	8	2	Sen = 66.7	35.4 – 88.7	>2.40	7	1	Sen = 63.6	31.6 – 87.6
≤5.07	4	3	Spec = 60.0	17.0 – 92.7	≤2.40	4	4	Spec = 80.0	29.9 – 98.9
			PPV = 80.0	44.2 – 96.5				PPV = 87.5	46.7 – 99.3
			NPV = 42.9	11.8 – 79.8				NPV = 50.0	17.4 – 82.6

Note: *D0* day of admission, *D3* third day and D8 - eighth day of hospitalization, *Sen* sensitivity, *Spec* specificity, *PPV* positive predictive value, *NPV* negative predictive value, *CI* confidence interval

## Discussion

Pneumonia is a disease associated to poverty conditions related to the environment, the individual, the infectious agent and the healthcare services [3, 4, 28–30]. In the present study, we adopted the criteria published in the Brazilian guidelines for community acquired pneumonia in pediatrics, which follows the former guidelines of WHO [3], allowing that patients (5–10% of the cases) with hidden pneumonia, which is characterized by fever and coughing, with or without radiographic changes, and improvement after empirical treatment, may be diagnosed at an early stage [1, 31, 32]. Thus, patients with fever, coughing, tachypnea and radiological changes were diagnosed with pneumonia, and those with subcostal recession were classified with severe pneumonia. In our casuistic, factors such age, anorexia, sibilance, difficult breathing, and vomiting were statistically correlated with pneumonia severity what corroborates with other studies [30, 33–36]. Presence of abdominal pain in children with pneumonia was observed by us as reported by other authors, but without any association with the disease severity [34, 37]. Anemia has a high prevalence in our midst, as demonstrated by Carvalho et al., who found a prevalence of 92% of healthy children in day care centers in the public health network with hemoglobin levels < 11.0 g/dL; but it was not also associated to severe pneumonia [12, 38]. The elapsed time between the beginning of the disease and its diagnostic, which can be related to the difficult access to the health services, was associated with disease severity, corroborating with other studies carried out in Brazil and Africa [33, 39]. Coincidently, all children under 5 years old were from countryside and presented severe pnuemonia. In this case, two aspects should be considered. First, a possible delay in diagnosis and treatment in the residence’s town may have contribute to an hemodynamic instability and ICU requirement at admission in the hospital; secondly, the innate immunity of young children may be compromised by less bactericidal activity and inflammatory response by immature neutrophils, and poor cytokine response by macrophages and monocytes [40].

Inflammation biomarkers, as cytokines, may be useful in determining the magnitude of the inflammatory response and lung injury in children with pneumonia. However, studies that tried to associate clinical severity in adult [18, 21, 41] and children [42] patients with respiratory diseases and serum levels of cytokines had controversial results. In the present study, the cytokines, TNF, IL-1β, IL-6, IL-8, IL-12p70, IFN-γ, IL-17A, IL-10 and IL-5, were detected in the serum of children with pneumonia and severe pneumonia at hospital admission, and IL-6 was the only cytokine associated with disease severity.

At admission (D0), we observed high serum levels of cytokines, predominantly pro-inflammatory including TNF, IL-1β, IL-6 and IL-8, which are involved in the innate immune response and in the immune response development [22, 43, 44]. In patients with severe pneumonia, the IL-6 levels were positively associated with high levels of leukocytes while the levels of TNF and IL-1β were associated with monocytes, possibly related to the recruitment and differentiation of macrophages at the site of inflammation. According to Bauer et al., TNF and IL-1β are associated with lung injury in acute respiratory distress syndrome and in severe pneumonia, although in a smaller magnitude [21]. A study reported by Kolsuz et al. on inflammatory cytokines (TNF, IL-1β, IL-6 and IL-8) levels in bronchoalveolar material after 24 h of admission for adult patients with pneumonia showed IL-6 as the most important cytokine in the determination of the disease severity in patients with systemic inflammatory response syndrome [45]. In other study, Antunes et al. reported high levels of IL-6, TNF-α, IL-10 and IL-1β detected in the majority of the 24 patients with CAP at admission, but the cytokines levels decreased significantly on days three and five of hospitalization, and IL-6 was the only cytokine associated with the disease severity, as ours findings [9].

High levels of IL-6 were observed in both studied groups at admission, highlighting the importance of this cytokine in the pulmonary inflammatory process regardless the disease severity [9]. Although IL-6 levels have been correlated with vomiting, which is a clinical manifestation of disease severity, other causes, such as the use of medications and inflammatory systemic process, may implicate in the etiology of vomiting and abdominal pain [34, 37]. IL-6 was also positively correlated with dyspnea, which may represent, under the point of view of the pathophysiology, the intensity of the lung’s inflammatory process, which is associated with respiratory disease severity.

An important anti-inflammatory cytokine in pneumonia is IL-10. In the present study, IL-10 levels were higher in the group of patients with pneumonia than in patients with severe pneumonia in admission (1.99 x 3.68 pg/mL; *P* = 0.074). Although the statistical differences were not significant what may be related to the small number of samples, the increased levels of IL-10 in patients with pneumonia was associated with high levels of lymphocytes, suggesting that IL-10 is an important modulatory factor of the inflammatory response of acute pneumonia [6, 24].

## Conclusion

In conclusion, the controversial results of some studies regarding the correlation of IL-6 serum levels and pneumonia severity may be produced by the choice of disease time point analysed and the balance between IL-6 and IL-10 serum levels. Our findings showed the ratio between the serum levels of IL-6 and IL-10 is an important criteria for severity definition at hospital admission, allowing to screening at the emergency room who is at risk of evolving into complication, and evaluation of recovery capacity. Specially in the third day of antibiotic therapy, when the patients’ clinical condition is usually evaluated for possible changing in the treatment protocol, an IL-6:IL-10 ratio higher than 5.0 may predict that 89% of positive cases have still severe pneumonia, indicating that changes in the treatment protocol might be needed. In future, the determination of IL-6 and IL-10 serum levels may be offered by routine laboratory as one more marker to evaluate the severity of infection disease at the diagnosis.

## Additional files

Additional file 1: Table S1. Clinical features of children with severe pneumonia and non-severe pneumonia. The differences between the clinic of children with severe and non-severe pneumonia included anorexia, wheezing, respiratory difficulty, subcostal recession, O2 saturation, but not respiratory or heart frequencies, fever or caughing; the duration of fever and hospitalization was longer in severe pneumonia. (DOC 75 kb)
Additional file 2: Table S2. Temporal evolution of clinical signs of patients according the pneumonia severity. Comparison of clinical signs inside the severe pneumonia group (Intragroup analysis) between different sampling times showed that changes in corporal temperature, O_2_ saturation and respiratory frequency were observed only in the eighth day (D8) of hospitalization; while in non-severe pneumonia group changes on corporal temperature and respiratory frequency were observed in the third day (D3) of hospitalization, confirming the delay recovery of severe pneumonia. Intergroup analysis corresponded to the comparison of clinical signs between severe and non-severe pneumonia groups in each time-point and showed significative differences in O_2_ saturation on D3 and D8 in relation to diagnosis (D0), as expected since it definies disease severity; differences in corporal temperature on D3 and respiratory frequency on D3 and D8 showed to be helpful to distinguish cases with unfavored evolution, since non-severe pneumonia recovery faster. (DOC 62 kb)

## Abbreviations

CAP: Community acquired pneumonia
CRP: C-reactive protein
ICU: Intensive care unit
IFNγ: Interferon gamma
IL: Interleukin
TNF: Tumor necrosis factor
WBC: White blood cell
WHO: World Health Organization
