# Supplementary material for: The balance between the serum levels of IL-6 and IL-10 cytokines discriminates mild and severe acute pneumonia
Source: BMC Pulm Med. 2016 Dec 1;16:170. doi: 10.1186/s12890-016-0324-z (PMC5131553; doi:10.1186/s12890-016-0324-z)
Supplement: Additional file 1: Table S1. — Clinical features of children with severe pneumonia and non-severe pneumonia. The differences between the clinic of children with severe and non-severe pneumonia included anorexia, wheezing, respiratory difficulty, subcostal recession, O2 saturation, but not respiratory or heart frequencies, fever or caughing; the duration of fever and hospitalization was longer in severe pneumonia. (DOC 75 kb) [file 12890_2016_324_MOESM1_ESM.doc]

**Supplementary Table 1** - Clinical features of children with severe pneumonia and non-severe pneumonia.

| **Clinical Variables** | **Severe pneumonia**  **(n=18)** | **Non-severe Pneumonia**  **(n=7)** | ***P-***  **value** |
| --- | --- | --- | --- |
| Age (years) |  |  |  |
| 0-4 | 9 (100 %) | 0 | **0.027** |
| > 4 | 9 (56.2 %) | 7 (43.8 %) |  |
| Gender |  |  |  |
| Male | 11 (78.6 %) | 3 (21.4 %) | 0.656 |
| Female | 7 (63.6 %) | 4 (36.4 %) |  |
| Coughing (days) |  |  |  |
| Median | 8 | 7 | 0.104 |
| Min –Max | 3-15 | 3-8 |  |
| Fever (days) |  |  |  |
| Median | 7 | 4 | 0.108 |
| Min –Max | 3-11 | 2-8 |  |
| Duration of fever during hospitalization  (days) |  |  |  |
| Median | 8 | 3 | **<0.001** |
| Min –Max | 4-29 | 2-5 |  |
| Anorexia (days) |  |  |  |
| Median | 6 | 5 | **0.023** |
| Min –Max | 4-16 | 2-7 |  |
| Wheezing |  |  |  |
| Yes | 16 (88.9 %) | 3 (42.8 %) | **0.032** |
| No | 2 (11.1 %) | 4 (57.2 %) |  |
| Vomiting |  |  |  |
| Yes | 5 (27.8 %) | 1 (14.3 %) | **0.002** |
| No | 13 (72.2 %) | 6 (85.7 %) |  |
| Respiratory difficulty |  |  |  |
| Yes | 16 (88.8 %) | 2 (28.6 %) | **0.007** |
| No | 2 (11.1 %) | 5 (71.4 %) |  |
| Subcostal recession |  |  |  |
| Yes | 18 (100 %) | 0 | **<0.001** |
| No | 0 | 7 (100 %) |  |
| O_2_ Saturation |  |  |  |
| Median | 94 | 98 | **0.011** |
| Min-Max | 85-98 | 96-98 |  |
| Respiratory Frequency |  |  |  |
| Median | 51 | 46 | 0.346 |
| Min -Max | 36 -80 | 38-52 |  |
| Heart frequency |  |  |  |
| Median | 120 | 115 | 0.394 |
| Min-Max | 100-160 | 102-144 |  |
| Chest Pain |  |  |  |
| Yes | 8 (44.4 %) | 1 (14.3 %) | 0.355 |
| No | 10 (55.6 %) | 6 (85.7 %) |  |
| Abdominal Pain |  |  |  |
| Yes | 11 (61.1 %) | 3 (42.8 %) | 0.656 |
| No | 7 (38.9 %) | 4 (57.2 %) |  |
| Anemia |  |  |  |
| Yes | 11 (61.1 %) | 4 (57.1 %) | 1.000 |
| No | 7 (38.9 %) | 3 (42.8 %) |  |
| Leukocyte Count |  |  |  |
| Median | 15,800 | 21,770 | 0.899 |
| Min –Max | (3,620-36,300) | (4,800-21,770) |  |
| Disease duration (days) |  |  |  |
| Median | 10 | 7 | **0.023** |
| Min –Max | 4-15 | 3-8 |  |
| Hospitalization (days) |  |  |  |
| Median | 16.5 | 4 | **<0.001** |
| Min –Max | 9-42 | 3-6 |  |
