# Supplementary material for: The balance between the serum levels of IL-6 and IL-10 cytokines discriminates mild and severe acute pneumonia
Source: BMC Pulm Med. 2016 Dec 1;16:170. doi: 10.1186/s12890-016-0324-z (PMC5131553; doi:10.1186/s12890-016-0324-z)
Supplement: Additional file 2: Table S2. — Temporal evolution of clinical signs of patients according the pneumonia severity. Comparison of clinical signs inside the severe pneumonia group (Intragroup analysis) between different sampling times showed that changes in corporal temperature, O2 saturation and respiratory frequency were observed only in the eighth day (D8) of hospitalization; while in non-severe pneumonia group changes on corporal temperature and respiratory frequency were observed in the third day (D3) of hospitalization, confirming the delay recovery of severe pneumonia. Intergroup analysis corresponded to the comparison of clinical signs between severe and non-severe pneumonia groups in each time-point and showed significative differences in O2 saturation on D3 and D8 in relation to diagnosis (D0), as expected since it definies disease severity; differences in corporal temperature on D3 and respiratory frequency on D3 and D8 showed to be helpful to distinguish cases with unfavored evolution, since non-severe pneumonia recovery faster. (DOC 62 kb) [file 12890_2016_324_MOESM2_ESM.doc]

**Supplementary Table 2** - Temporal evolution of clinical signs of patients according the pneumonia severity.

| **Severe Pneumonia** | | | | | |  | **Non-severe Pneumonia** | | | | |  | **Severe x Non-severe Pneumonia** | |
| --- | --- | --- | --- | --- | --- | --- | --- | --- | --- | --- | --- | --- | --- | --- |
| **Variables** | **Minimum** | **Maximum** | **Median** | **Comparison groups** | ****P*-value** |  | **Minimum** | **Maximum** | **Median** | **Comparison groups** | *****P*-value** |  | **Comparison groups** | ******P*-value** |
| SAT O_2_ (D0) | 85 | 98 | 94 | D0xD3 | 0.120 |  | 96 | 98 | 98 | D0xD3 | 0.121 |  | D0xD0 | **0.011** |
| SATO_2_ (D3) | 90 | 99 | 97 | D3xD8 | **0.013** |  | 98 | 99 | 98 | D3xD8 | **0.034** |  | D3xD3 | **0.021** |
| SATO_2_ (D8) | 94 | 99 | 98 | D0xD8 | **0.000** |  | 98 | 99 | 99 | D0xD8 | **0.007** |  | D8xD8 | 0.056 |
|  |  |  |  |  |  |  |  |  |  |  |  |  |  |  |
| TEMP (D0) | 38 | 39.2 | 38.4 | D0xD3 | 0.393 |  | 38 | 39 | 38.3 | D0xD3 | **0.002** |  | D0xD0 | 0.629 |
| TEMP (D3) | 37.5 | 39.5 | 38 | D3xD8 | **0.001** |  | 36 | 37.9 | 37 | D3xD8 | 0.479 |  | D3xD3 | **0.000** |
| TEMP (D8) | 36.5 | 38.5 | 37 | D0xD8 | **<0.000** |  | 36 | 37 | 37 | D0xD8 | **0.002** |  | D8xD8 | 0.053 |
|  |  |  |  |  |  |  |  |  |  |  |  |  |  |  |
| RF (D0) | 38 | 80 | 51 | D0xD3 | 0.162 |  | 38 | 52 | 46 | D0xD3 | **0.012** |  | D0xD0 | 0.346 |
| RF (D3) | 27 | 64 | 46 | D3xD8 | **0.014** |  | 32 | 42 | 36 | D3xD8 | **0.020** |  | D3xD3 | **0.030** |
| RF (D8) | 28 | 48 | 38 | D0xD8 | **0.001** |  | 24 | 36 | 30 | D0xD8 | **0.003** |  | D8xD8 | **0.008** |
|  |  |  |  |  |  |  |  |  |  |  |  |  |  |  |
| CF (D0) | 110 | 160 | 120 | D0xD3 | 0.408 |  | 102 | 144 | 115 | D0xD3 | 0.100 |  | D0xD0 | 0.394 |
| CF (D3) | 87 | 160 | 116 | D3xD8 | 0.253 |  | 89 | 115 | 107 | D3xD8 | 0.810 |  | D3xD3 | 0.141 |
| CF (D8) | 100 | 140 | 110 | D0xD8 | 0.055 |  | 98 | 120 | 102 | D0xD8 | 0.073 |  | D8xD8 | 0.390 |

Note: RF - respiratory frequency; CF- cardiac frequency; TEMP - body temperature; SAT O_2_ - oxygen saturation; D0 - day of admission; D3- third day and D8 - eighth day of hospitalization. Intragroup analysis consisted in comparison of clinical signs between different sampling times inside each studied group: severe pneumonia group (**P*-value) and pneumonia group (***P*-value). Intergroup analysis corresponded to the comparison of clinical signs between severe and non-severe pneumonia groups in each time point (****P*-value).
